# Psychosocial Interventions for Women with a *BRCA1* or *BRCA2* Mutation: A Scoping Review

**DOI:** 10.3390/cancers13071486

**Published:** 2021-03-24

**Authors:** Talin Boghosian, Jeanna M. McCuaig, Lindsay Carlsson, Kelly A. Metcalfe

**Affiliations:** 1 Women’s College Research Institute, Women’s College Hospital, Toronto, ON M5G 1N8, Canada; tboghosian@ovariancanada.ca; 2 Lawrence S. Bloomberg Faculty of Nursing, University of Toronto, Toronto, ON M5T 1P8, Canada; Jeanna.McCuaig@uhn.ca (J.M.M.); Lindsay.Carlsson@uhn.ca (L.C.); 3 Familial Cancer Clinic, Princess Margaret Cancer Centre, University Health Network, Toronto, ON M5G 2M9, Canada; 4 Department of Molecular Genetics, University of Toronto, Toronto, ON M5T 3A9, Canada

**Keywords:** psychosocial interventions, BRCA mutation, support, support groups, peer support

## Abstract

**Simple Summary:**

Women with a *BRCA1* or *BRCA2* mutation are at an increased risk of developing hereditary breast and ovarian cancers. While genetic counselling by genetic counsellors takes place before and after receiving the results of genetic testing, genetic counsellors are not involved in the patient’s long-term psychosocial follow-up. Genetic testing can cause short-term and long-term distress in women with a *BRCA1* or *BRCA2* mutation, and follow-up supports may be necessary for some women. As the uptake of genetic testing for hereditary breast and ovarian cancer increases, the need for additional sources of support may be needed. This review examined the effectiveness of psychological and psychoeducational interventions for *BRCA* mutation carriers.

**Abstract:**

This scoping review aimed to explore the effectiveness of psychological and psychoeducational interventions for *BRCA* mutation carriers. Four electronic bibliographic databases were searched. After review, 23 articles that described or assessed forms of an additional psychosocial intervention for individuals with a *BRCA* mutation were identified and included. Intervention types discussed in the articles were telephone-based peer-to-peer counselling (5), online communities (4), in-person group counselling (8), and one-day sessions (6). Outcomes investigated within the articles included psychosocial outcomes (18), satisfaction (8), health behaviours (7), and knowledge (5). The included studies suggested that telephone-based peer-to-peer counselling and online communities improve patient knowledge and psychosocial functioning and can overcome challenges such as scheduling and travel associated with in-person support groups, but may have challenges with recruitment and retainment of participants. Group in-person education sessions satisfied the need amongst *BRCA1/2* carriers in terms of accessing necessary information regarding cancer risk assessment and management; however, the impact of group education sessions on psychological outcomes was variable across the included studies. Overall, all the forms of intervention described in this scoping review were well-received by participants; some have been shown to reduce distress, depression, and anxiety.

## 1. Introduction

Within the general population, the risk of carrying a *BRCA1* or *BRCA2* mutation is approximately 1 in 400 [1]. Women who carry pathogenic mutations in *BRCA1* and *BRCA2* have an increased risk of developing hereditary breast and ovarian cancers (HBOC) [2]. Previous research has shown that some women may experience short and long-term distress when they learn that they carry a *BRCA1* or *BRCA2* mutation [3,4,5]. Though distress often decreases over time, some women have elevated levels of distress in the long-term [6,7,8,9]. We have previously reported on the prevalence of long-term distress in an international cohort of women with a *BRCA1* or *BRCA2* mutation [10]. At an average of 5 years post genetic testing, 16.3% of the women had moderate to severe cancer-related distress. Some women with a *BRCA1* or *BRCA2* mutation may benefit from both short and long-term psychosocial follow-up. However, psychosocial follow-up is not often standard of care.

Most often, women undergoing genetic testing receive genetic counselling to discuss cancer risks and management strategies. Genetic counselling is provided by a genetic counsellor and generally provided prior to genetic testing to ensure that a woman is fully informed of the risks and benefits of genetic testing and after testing, to disclose test results and provide management recommendations. Genetic counsellors are not typically involved in the long-term care of a patient. As the interest in genetic testing for HBOC expands, alternative models of genetic testing are being used globally [11]. Some of these models of care have very limited interactions with a genetic counsellor, and as a result, the psychosocial functioning of a patient after receiving a positive genetic test result may not be adequately assessed. We have recently reported that some of the alternative models of care (such as telephone, telegenetic, group genetic counselling, or models with no pre-test genetic counselling) may not be appropriate for women who require additional emotional support, and yet these models are increasingly used in clinical practice [11].

It is evident that additional psychosocial support may be necessary for some patients after receiving a positive genetic test result. The purpose of this review is to provide a single comprehensive review of the effectiveness of psychological and psychoeducational interventions for *BRCA* mutation carriers, which can serve to guide their use and development as access to genetic testing continues to expand.

## 2. Materials and Methods

The methodology that was used for this review was based on the Preferred Reporting Items for Systematic Reviews and Meta-analyses Extension for Scoping Reviews (PRISMA-ScR) Checklist, Supplemental Digital Content A, as well as the framework outlined by Levac et al. (2010) [12,13]. The protocol was not registered.

### 2.1. Search Strategy

A systematic search was conducted in four electronic bibliographic databases, PsycINFO, CINAHL, MEDLINE, and EMBASE, for studies published from database initiation to May 2020. An initial search was conducted on 6 April 2020, and an updated search was completed on 29 May 2020. MeSH headings and keywords used in the search included: *BRCA mutation, support groups, peer support,* and *psychosocial interventions* (Search Strategy and Dates, Supplemental Digital Content B. Relevant articles were identified in advance and used to validate the search strategy.

### 2.2. Eligibility Criteria

Studies were eligible for inclusion if they described or assessed forms of an additional psychosocial intervention for individuals with a *BRCA* mutation. The additional psychosocial intervention was defined as support received outside of a traditional genetic counselling session or medical intervention, either in person, by telephone, or in a virtual space (e.g., social media groups, chat rooms, or web-based discussion boards) from an individual or group of individuals [14]. Articles were included if the individuals receiving an intervention were carriers of a *BRCA1* or *BRCA2* mutation. All types of research design were included. Only articles published in the English language were included. Conference abstracts, dissertations, and non-profit reports were included.

### 2.3. Study Selection

All abstracts were screened for inclusion by two reviewers blinded to each other’s judgements (J.M.M. and T.B.). If concordance was not reached on abstract review, the disagreement was resolved through discussion. A full-text review was conducted by two reviewers (J.M.M. and T.B.) independently to determine final eligibility. Conflicts in eligibility were resolved through discussion. References of articles selected following full-text review were screened to identify additional articles.

### 2.4. Data Extraction and Analysis

Data charting and extraction was performed by T.B. Using a data extraction table, information was collected on research design, study population, psychosocial intervention characteristics, participant outcomes. Articles were grouped by intervention type.

## 3. Results

### 3.1. Search Results

**Figure 1:**
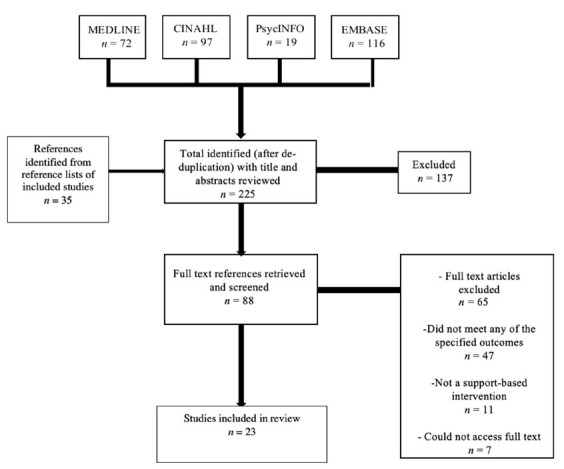
PRISMA flow chart of included studies.

The initial search resulted in 225 publications. Following title and abstract screening, 88 publications went through to full-text review. Twenty-three papers met eligibility criteria and were included in this review (Figure 1).

### 3.2. Characteristics of Included Studies

Of the 23 publications included, two were review articles, and 21 were full-text articles. Studies were published between 2004 and 2019. Study designs included randomized controlled trials (3), qualitative content analysis (18), non-randomized cohort study approach (1), and mixed-method (1) approaches. Recruitment of participants occurred through familial cancer clinics (18), and web-based platforms (4), such as the website Facing Our Risk of Cancer Empowered (FORCE) (https://www.facingourrisk.org/ (accessed on 20 June 2020)). Studies were grouped into four categories based on intervention type: telephone-based peer-to-peer counselling (5), online communities (4), in-person group counselling (8), and one-day information day (6) (Table 1, Table 2, Table 3 and Table 4). The most common outcomes were psychosocial outcomes (18), satisfaction (8), health behaviours (7), and knowledge (5).

### 3.3. Telephone Based Intervention

Telephone peer-to-peer matching models involved two *BRCA* mutation carriers matched with another *BRCA* mutation carrier who underwent a training course. Five articles were identified, four of which had a peer (a woman with an identified *BRCA* mutation as well) as the support provider, and three of those articles mentioned the selected peers undergoing training. The remaining study involved the provision of support by a genetic counsellor or mental health counsellor.

In assessments of satisfaction, most peers and recipients of the intervention were very satisfied [15,16,17,18]. Qualitative analyses found that peers reported feeling valuable given the support they were able to provide, while recipients reported a reduced sense of isolation and the usefulness of hearing the experiences and advice of their peers [15,17]. Recipients reported a decrease in decisional conflict and an increase in healthy coping strategies, such as planning and positive reframing [18]. However, one study found that the dropout rate of participation was higher in intervention groups than the usual care group, who did not receive any intervention [19]. One study reported mixed views about the telephone as the mode of intervention, with some participants reporting being more comfortable with it while others reported it as a barrier to non-verbal communication [15]. When possible, the studies had aimed to match peers and recipients based on medical history and age, and more specific matching (regarding things like the intention to have children) was suggested as an area of improvement [17].

In studies assessing psychosocial outcomes, levels of anxiety (related to *BRCA* mutation status), breast cancer distress, and depression were significantly lower after participation in the intervention [19,20]. White et al. also reported a reduction in cognitive appraisals about genetic testing stress as well as a lower score in unmet information needs among participants who underwent intervention, compared to the group who did not receive intervention [19].

**Table 1:** Telephone-based intervention.

Study	Country	Population	Study Design	Outcomes	Results
White et al. (2014) [19]	AUS	Women with *BRCA* mutation receiving telephone intervention (*n*= 102), no intervention (*n* = 102), and peers (*n* = 41).	Randomized controlled trial	Satisfaction Psychosocial	There was a significantly lower level of stress in the IG. Cognitive appraisals scores for stress were reduced in the IG at time two, but the difference was not significant at time three IG group had statistically significant lower mean anxiety at time two, reduced to nonsignificant at time three. Unmet information needs were lower in the IG at time two, and the difference was not significant at time three.
St. Pierre et al. (2018) [15]	CAN	Peer intervention among women with *BRCA* mutations who were considering prophylactic mastectomy (recipients, *n* = 15) and women who had undergone this surgery (peers, *n*= 19).	Cross-sectional study	Satisfaction Psychosocial	Peers were satisfied with the support they provided. Peers had mixed views about the value of the telephone as the mode of intervention 100% of recipients found the telephone-based intervention useful in their consideration of prophylactic mastectomy. Recipients reported a reduced sense of isolation.
Farrelly et al. (2015) [17]	AUS	A qualitative study of a peer-support program for women aged 29–71, consisting of recipients with a *BRCA* mutation (*n* = 105) and peers (*n* = 37)	Cross-sectional study	Satisfaction Psychosocial	Seventy-eight percent of peers were satisfied with their experience. Ninety-one percent of peers said providing support made them feel valuable. Eighty-seven percent of recipients were satisfied. Ninety-two percent of recipients said their peer made them feel less alone.
Graves et al. (2010) [20]	USA and CAN	Women with a *BRCA* mutation receiving genetic counselling and a telephone intervention (*n* = 47) or just traditional genetic counselling (*n* = 43)	Randomized trial	Psychosocial	The IG reported significantly lower depression scores at six months compared with the traditional group. The IG was less likely to report clinically significant levels of anxiety, depression, and overall distress at six months. Women in the intervention group reported reduced genetic testing distress at six months.
O’Neill et al. (2018) [18]	USA	Pilot intervention assessed the information needs and distress levels in women with a *BRCA* mutation (*n* = 100)	Cross-sectional study	Satisfaction Psychosocial	Prior to intervention, found 63% of participants had unmet information needs. Intervention resulted in a significant decrease in decisional conflict and a significant increase in healthy coping strategies.

IG = intervention group; UCG = usual care group; OC = ovarian cancer; BC = breast cancer; HBOC = hereditary breast/ovarian cancer.

### 3.4. Online Communities

Four publications described online communities such as Facing Our Risk of Cancer Empowered (FORCE) (www.facingourrisk.org (accessed on 20 June 2020)) or communities hosted on pages like Facebook. These are often not moderated; rather, they serve as a space for individuals with *BRCA* mutations to discuss the challenges they face, exchange information, and provide social support. The articles, qualitative in nature, describe group members sharing experiences, concerns and choices with each other [21,22]. The platform serves as a way for many women to connect with others outside of their own family members who were also *BRCA* mutation carriers and provided an opportunity to speak to others with a similar experience [22].

Online communities served as a valuable source of information, as Hesse-Biber et al. reported that in the proceeding six months after a positive test result, the more engaged and involved a woman was with her online *BRCA* community, the more likely it was she would have risk-reducing surgery [23]. Dean et al. reported that many of the women in the FORCE community had discovered the online community while seeking out referrals for social support from their health care providers [24].

**Table 2:** Web-Based Intervention.

Study	Country	Population	Study Design	Outcomes	Results
Stefansdottir (2016) [21]	ISL	Female Icelandic *BRCA* mutation carriers	Cross-sectional study	Psychosocial	Members of the group share experiences, concerns, good and bad news, joy and choices with each other.
Kenen et al. (2007) [22]	USA	An observational study of women with a *BRCA* mutation (*n* = 29) who had not had cancer (*n* = 17) or were cancer survivors (*n* = 5)	Cross-sectional study	Psychosocial	The social community built online allowed for reducing a sense of isolation. Online community functioned as a source of information and a means for communal problem-solving.
Dean et al. (2018) [24]	USA	Women with *BRCA* mutations (*n* = 34) aged 27–67 who do not have cancer and participate in an online chatroom	Cross-sectional study	Psychosocial Health Behaviours	Women sought out referrals from health care providers for social support. Women discussing their fears and concerns about their high genetic risk helped them manage their uncertainties.
Hesse-Biber et al. (2016) [23]	USA	A qualitative study of women with *BRCA* mutations (*n* = 303)	Cross-sectional study	Health Behaviours	The more uncertain a women felt about familial support, the less likely it was she would decide to have risk-reducing surgery, and more likely she would continue surveillance. The more involved a participant was with the *BRCA* community months after the test, the more likely she would decide to have risk-reducing surgery than to continue surveillance.

### 3.5. Group In-Person

In-person support groups were moderated by trained professionals such as genetic counsellors, social workers, and nurses. Eight articles were identified which involved recurring, in-person group sessions. Within the publications, group sizes varied between 4–12 individuals, three occurring weekly and one yearly. Four of the studies mentioned an emphasis on the educational component of the group intervention.

Six articles assessed satisfaction, and all reported high levels of satisfaction among participants who underwent the intervention [25,26,27,28,29,30]. Only Visser et al. observed a preference for individual visits over group medical visits (by participants and medical professionals); however, this may be attributed to the group visit being in lieu of an individual medical visit, rather than an additional visit [26]. Those who participated in the Visser et al. study did report satisfaction related to the peer support component of the group medical visit, indicating not feeling alone and appreciating the advice of other participants [26]. Four other studies also evaluated support and found participants experienced emotional support and a diminished level of isolation [27,28,29,30,31].

Four studies assessed knowledge, and while three found increased medical knowledge of cancer surveillance and risk-reducing surgery options, one found the increase was not statistically significant [25,28,29,31].

Regarding health behaviours, Landsbergen et al. reported that while risk-reducing management and surveillance preferences did not change in participants of the intervention or in the usual care group; however, women who had indicated a preference for mastectomy were more inclined to get one after attending the interventions [28].

The four articles that assessed psychosocial factors reported reductions in anxiety, depression, or cancer worry [27,29,31,32].

**Table 3:** Group In-person Intervention.

Study	Country	Population	Study Design	Outcomes	Results
Myklebust et al. (2016) [25]	NOR	An observational study of women with a *BRCA* mutation (*n* = 17) who attended an educational support group	Cross-sectional study	Psychosocial Satisfaction Patient Preferences Knowledge Health Behaviours	Participants had received different information from various doctors and were left feeling frustrated with healthcare professionals they felt lacked insight into the patient’s point of view. Participants were pleased to have received consistent information from healthcare professionals during ESG. Reasons for attending ESG were to gain insight on the experience of other mutation carriers, need for information, need for emotional support. Following the ESG, several participants had become more conscious of their choices regarding risk-reducing surgeries.
Visser et al. (2011) [26]	NLD	A randomized pilot study of patients with *BRCA* mutations (*n* = 7) attending a group medical consultation	Randomized trial	Satisfaction Psychosocial	The average overall satisfaction score was 3.7/5; 57% would choose a GMC again, and 42% experienced support from others.
Visser et al. (2016) [27]	NLD	Randomized study of women aged 25–60 with *BRCA* mutations attending a group medical consultation (*n* = 63) and traditional appointments (*n* = 59)	Randomized trial	Satisfaction Psychosocial Health Behaviours Patient Preferences	Groups did not significantly differ on distress. The frequency of breast self-examination did not significantly differ. Participants and healthcare professionals were less satisfied with GMCs than individual visits. Participant satisfaction was positively related to the experience of peer support.
Landsbergen et al. (2010) [28]	NLD	A qualitative study of women with *BRCA* mutations (*n* = 34) attending educational support group led by a social worker	Cross-sectional study	Satisfaction Knowledge	Participants indicated that group participation highly met their needs of *BRCA*-related information to support their decision-making regarding cancer surveillance or prophylactic surgery.
Esplen et al. (2004) [29]	CAN and AUS	A non-randomized intervention of women with *BRCA* mutation (*n* = 70)	Non-randomized trial	Satisfaction Psychosocial Knowledge Health Behaviours	Significant improvements were observed in cancer worries, anxiety, and depression. The intervention reduced the intensity of grief for those who had their genetic test result <1 year compared to those who had the result for >1 year. For those who had their genetic test result for >1 year, the intervention reduced levels of intrusion/cancer worry. An increase in general health-related quality of life was not statistically significant. Breast cancer risk knowledge improvement and was not statistically significant. No significant changes in rates of clinical breast examinations; self-reported improvement in feelings of anxiety around BSE. Ninety-seven percent of participants reported a very high level of satisfaction. A significant number of women made decisions concerning prophylactic surgery during and after the intervention.
Listøl et al. (2017) [32]	NOR	Women with a *BRCA* mutation (*n* = 100) who attended a group-based patient education course	Cohort study	Psychosocial	Significant decrease in anxiety at time two. Depression scores did not change significantly from time one to time two.
Mendes et al. (2010) [30]	PRT	A qualitative study of women aged 24–74 with a *BRCA* mutation (*n* = 9)	Cross-sectional study	Satisfaction Psychosocial	A group experience of sharing removed the inadequacy of some feelings and thoughts and prevented isolation. All participants felt the informative session was very important, allowing an effective integration of medical information. Increased self-assurance when considering decision making about undergoing prophylactic surgery and improved confidence to undergo risk reduction procedures.
Ducaine et al. (2009) [31]	NR	Participants aged 40–55 with a *BRCA* mutation who attended a hospital-based focused support group	Cross-sectional study	Psychosocial Knowledge	Eighty-five percent of participants felt that attending the support group significantly improved their medical knowledge. Seventy percent of participants felt that the group provided significant emotional support. Ninty-six percent of participants felt they were better able to broach the subject of their *BRCA* status with family members. Fifty-six percent of participants experienced significantly lowered anxiety levels regarding their *BRCA* status Seventy-eight percent of participants reported diminished feelings of isolation.

ESG = education support group; GMC = group medical consultation; BSE = breast self-examination.

### 3.6. One-Day Session

Five publications described one-day interventions: these ranged from more casual retreats to more structured information sessions to a conference with seminars, teleconferences, and webinars. Discussion topics included general information about hereditary breast and ovarian cancers, risk-reducing surgeries and surveillance, and body image and sexual health [16,32,33,34,35].

Two studies reported a decrease in anxiety and psychological distress, and one study observed no significant change [32,33,34]. Participants in one-day sessions reported feeling less isolated, more empowered, and reported high satisfaction with the intervention [16,33,35]. McKinnon et al. found half of their participants that altered their health behaviour did so as a result of the intervention; this included increasing cancer screening and completing or considering surgery [34].

**Table 4:** One-Day Session.

Study	Country	Population	Study Design	Outcomes	Results
Harris et al. (2011) [35]	GBR	Women aged 29–58 with a *BRCA* mutation (*n* = 33) who attended a one-day information and psychological support forum	Cross-sectional study	Psychosocial Knowledge	Ninty-seven percent of participants reported that the forum gave them a sense of belonging and that they valued the opportunity to share their experiences, fears, and anxieties in small groups. Eighty-three percent of participants agreed they had a better understanding of being a *BRCA* gene carrier and the options available to them. Twenty-three percent of participants described the day as having given them a sense of empowerment.
McKinnon et al. (2007) [34]	USA	A qualitative study of participants with *BRCA* mutations (*n* = 41) attending a one-day retreat	Cohort study	Health Behaviours	Fifty percent of participants that completed both sets of questionnaires made changes in their lifestyle (exercising, reduced fat intake, etc.) following the retreat, and over half said it was due to information they received at the retreat. Forty-two percent of participants who completed both sets of questionnaires reported that they increased their cancer screening, initiated chemoprevention, completed or are considering prophylactic surgery due to information they received at the retreat.
Bober et al. (2015) [33]	USA	Women aged 36.8–49.7 with *BRCA* mutations who previously underwent RRSO (*n* = 37)	Cross-sectional study	Psychosocial Satisfaction Knowledge	Overall sexual functioning, desire, arousal, satisfaction, and pain improved significantly. Significant reductions in somatization and anxiety scores. Knowledge about sexual side effects of RRSO significantly improved from baseline to postintervention. Participants were highly satisfied with the intervention and reported utilizing new skills to manage sexual dysfunction.
Landsbergen et al. (2010) [28]	NLD	An observational study of women with a *BRCA* mutation but not a personal history of breast cancer and/or ovarian cancer (*n* = 163)	Cohort study	Health Behaviours	ESG participants were less likely to opt for *BRCA* surveillance but were more likely to prefer mastectomy than non-participants. Attending an ESG did not change the prior preference of *BRCA* surveillance or prophylactic mastectomy. Among women with an intention to undergo prophylactic mastectomy, those who attended an ESG were significantly more inclined to have the surgery than women who did not attend.
Listøl et al. (2017) [32]	NOR	Women with *BRCA* mutations (baseline *n* = 100, post-intervention *n* = 75) who attended a group-based patient education course	Cohort study	Psychosocial	Anxiety scores significantly decreased from time one to time two. Depression scores did not change significantly from time one to time two.
Tercyak et al. (2015) [16]	USA	Jewish women with at risk for and/or living with breast and/or ovarian cancer (*n* = 133)	Cross-sectional study	Patient Preferences	Among six resources offered by the Sharsheret organization, the highest level of engagement was with the peer support network and health care symposia.

RRSO = risk-reducing salpingo-oophorectomy, ESG = education support group.

## 4. Discussion

This scoping review provides initial insights into patient preferences and psychosocial outcomes associated with alternate models of post-disclosure support interventions. While the mode of intervention differed, the focus of all of the interventions was directed towards enhancing knowledge of hereditary breast and ovarian cancer and providing emotional support. Many women reported unmet needs related to the information provided by primary health providers and support offered by family members following result disclosure. The educational and supportive elements built into these interventions empowered women to cope with their known genetic risk and make informed health-related decisions.

Telephone peer-to-peer interventions included in this review were associated with favourable outcomes for recipients. Peer coaching offers *BRCA* mutation carriers both the experiential understanding and informational expertise necessary to guide their decision-making and to offer psychological support [36]. Findings from qualitative studies in this review demonstrated positive recipient perceptions associated with the telephone peer support, including high satisfaction rates and perceived utility of the intervention.

Peer support interventions have been studied across various types of cancers and in response to different clinical needs, such as improving access to clinical services and supporting cancer patients during treatment [37,38,39]. Similar to the findings in this review, peer support or coaching interventions were associated with high levels of satisfaction and perceived utility in breast cancer patients and women from HBOC families [36,38].

Like other studies, this review also reinforced the importance of the peer matching process and those variables that are critical to the success of peer support, including gender, age, and personal cancer status [36,37]. Given the high perceived benefits and clinical utility of this intervention, future studies should evaluate the necessary content and training requirements for peer mentors such that this intervention can be standardized and implemented more broadly across clinical communities.

Summarizing the psychological outcomes secondary to telephone peer support was challenging in this review as different assessment tools were used, ranging from single questions to psychometrically validated tools. White and colleagues (2014) used the Impact of Event Scale to measure breast cancer distress and reported lower levels of distress in the telephone to peer support intervention group compared to the usual care group. Although the levels of breast cancer distress were statistically lower in the intervention group, the mean value remained below the clinically significant threshold in both groups (<25 on the Impact of Events Scale (IES) tool) [19].

Conversely, Graves and colleagues (2010) demonstrated a significant reduction in decisional conflict in their pilot telephone peer support program [20]. Although there was a significant reduction noted in decisional conflict, the post-intervention level still demonstrated moderate conflict. Decisional conflict is a key outcome of interest in the literature evaluating decision aids for *BRCA* mutation carriers and studies comparing different modes of pre-testing genetic counselling [40,41]. Thus, decisional conflict may be an important outcome in future studies evaluating telephone peer-to-peer programs, especially those programs aiming to support women in their health-related decision.

Online communities offer social support at a safe distance, as these open forums provide anonymity and convenient access to other individuals with shared experiences [42]. Such forums can offer emotional and informational support, as well as feedback and reassurance related to one’s health-related decisions. Online support usage amongst individuals diagnosed with cancer is quite variable [43]. Seeking out online communities is often secondary to an unmet need or dissatisfaction with informational support provided by their clinical team, especially in relation to treatment decisions and/or symptom management [43]. This overlaps with the findings in this review, where recipients valued engaging with other women known to harbour a *BRCA* mutation, specifically regarding their decisions about cancer prevention and screening. This engagement reduced feelings of isolation and allowed women to access support not offered through their clinical team or family members.

Studies in this review suggest telephone and online communities improve patient knowledge and psychosocial functioning. They are also able to circumvent the challenges of scheduling and the necessity of travel required in in-person support groups and one-day sessions. However, the data also suggests challenges with recruiting and retaining participants with online interventions. Further research is needed to identify those individual characteristics that influence one’s preferences towards participating in virtual versus in-person modes of support.

In-person group education sessions responded to a clear unmet need amongst *BRCA* mutation carriers in terms of accessing necessary information regarding cancer risk assessment and management. Primary care providers are often unaware of available cancer screening and prevention recommendations [44], which can contribute to feelings of frustration and anxiety amongst patients. Group sessions led by health care providers were found to increase knowledge levels and perceived sense of support. Previous research on Lynch syndrome has also demonstrated that participants in group support sessions report high levels of satisfaction and that the content met their information and emotional support needs [45]. This echoes the findings in this review, where women described feeling knowledgeable and confident in their decisions surrounding cancer prevention, as well as feeling a sense of inclusion.

The impact of group education sessions on psychological outcomes was variable across studies in this review. Esplen and colleagues (2004) described a reduction in distress, anxiety and depression following a 12-week supportive-expressive group therapy intervention [29], whereas Visser and colleagues (2016) did not demonstrate any change in psychological outcomes following group medical consultations [27]. The latter reflects previous research evaluating a group pre- and post-test counselling and education intervention [46]. These variable findings suggest the content and structure of the group sessions may impact the psychological outcomes of participants, and thus further research is needed to evaluate the design and implementation of these interventions. Randomized trials evaluating the impact of peer support on the quality of life of breast cancer patients have similarly identified variability in peer selection and implementation of the peer support program as contributing to the variability in their findings [47,48].

Some limitations to the current review should be noted. First, publications selected for the review comprise of only empirical papers published in academic, peer-reviewed journals, published in English. Second, many of the publications identified did not have control or comparison groups, and outcome measures were qualitatively assessed without validated outcome measures. Considering this methodological weakness in some of the included studies, the articles still provide valuable insights into psychosocial interventions available in conjunction with traditional support. Third, some of the included articles describe pilot studies where the interpretation of measured outcomes is limited by a small sample size. Fourth, a quality assessment was not completed for the included articles. The overall review likely had fair generalizability given the variety of recruitment methods and studies recruiting an under-represented population, but the generalizability of any given finding is uncertain.

Overall, psychological and psychoeducational interventions for women with a *BRCA1* or *BRCA2* mutation are well-received by participants, and some have been shown to reduce distress, depression, and anxiety. Although some of the studies reviewed reported no significant psychosocial benefits, it should be noted that none of the interventions reviewed reported negative consequences. These interventions may provide needed support to women, as new models of genetic testing are introduced into clinical practice where access to a genetic counsellor is limited.

## Data Availability

Not applicable.

## Supplementary Materials

The following are available online at https://www.mdpi.com/2072-6694/13/7/1486/s1.
