# Supplementary material for: Psychosocial Interventions for Women with a BRCA1 or BRCA2 Mutation: A Scoping Review
Source: Cancers (Basel). 2021 Mar 24;13(7):1486. doi: 10.3390/cancers13071486 (PMC8037801; doi:10.3390/cancers13071486)
Supplement: Supplementary file 1 [file cancers-13-01486-s001.zip › Supplemetal B.docx]

**Supplemental Content 1: Search Strategy & Dates for Scoping Review of Support Interventions for Women with *BRCA1/2* Mutations**

**CINAHL**: 97

April 6, 2020

( BRCA or BRCA1/2 or BRCA1 or BRCA2 or hereditary ) AND ( support groups or peer support or group support or group interventions or psychosocial ) AND ( online or web or internet or distance or video* or virtual or *phone or tele* or e-* )

### EMBASE: 116

April 6, 2020

BRCA or BRCA1/2 or BRCA1 or BRCA2 or hereditary in Title Abstract Keyword AND internet or online or web or virtual or e* or tele* or video* or virtual or phone or technology or computer in Title Abstract Keyword AND support group or peer support or *social support* or group intervention or pyschosocial

[**PsychINFO**](http://search.ebscohost.com/login.aspx?direct=true&AuthType=ip,uid&db=psyh&bquery=(carer+OR+caregiver+OR+(family+AND+carer)+OR+caregiving)+AND+((support+AND+group)+OR+(peer+AND+support)+OR+(group+AND+support)+OR+(group+AND+intervention))+AND+(online+OR+web+OR+internet+OR+distance+OR+video+OR+virtual+OR+(phone+AND+for)+OR+tele+OR+video)+AND+(dementia+OR+alzheimers+OR+(cognitive+AND+impairment)+OR+(memory+AND+loss)+OR+(cognitive+AND+decline)+OR+(mild+AND+cognitive+AND+impairment)+OR+Parkinson)+NOT+((social+AND+media)+OR+(adaptive+AND+technology))&type=1&site=ehost-live): 19

April 6, 2020

(BRCA or BRCA1/2 or BRCA1 or BRCA2) AND (support group or peer support or group support or group intervention ) AND ( online or web or internet or distance or video or virtual or phone for or tele or video )

**MEDLINE**: 72

April 6, 2020

(((((((((English[Language]) AND (BRCA or BRCA1/2 or BRCA1 or BRCA2 or hereditary[MeSH Terms])) AND (support group or peer support or group support or group intervention[MeSH Terms])) AND (online or web or internet or distance or video* or virtual or *phone or tele* or e-*[MeSH Terms])))
